# Necrotizing Fasciitis in Paroxysmal Nocturnal Hemoglobinuria

**DOI:** 10.1155/2015/908087

**Published:** 2015-08-11

**Authors:** Pusem Patir, Yakup Isik, Yigit Turk, Mehmet Can Ugur, Cengiz Ceylan, Gulnur Gorgun, Nihal Mete Gokmen, Guray Saydam, Fahri Sahin

**Affiliations:** ^1^Department of Hematology, Ege University, 35100 Izmir, Turkey; ^2^Department of Plastic and Reconstructive Surgery, Ege University, 35100 Izmir, Turkey; ^3^Department of General Surgery, Ege University, 35100 Izmir, Turkey; ^4^Department of Internal Medicine, Tepecik Education and Training Hospital, Izmir, Turkey; ^5^Department of Immunology, Ege University, 35100 Izmir, Turkey

## Abstract

Paroxysmal nocturnal hemoglobinuria (PNH) is a rare, progressive, and life-threatening hematopoietic stem cell disorder characterized by complement-mediated intravascular hemolysis and a prothrombotic state. Patients with PNH might have slightly increased risk of infections due to complement-associated defects subsequent to CD59 deficiency. Here, we report a rare case of a 65-year-old male patient with necrotic ulcers on both legs, where the recognition of pancytopenia and microthrombi led to the diagnosis of PNH based on FLAER (FLuorescent AERolysin) flow cytometric analysis. He was subsequently started on eculizumab therapy, with starting and maintenance doses set as per drug labelling. Progression of the patient's leg ulcers during follow-up, with fulminant tissue destruction, purulent discharge, and necrotic patches, led to a later diagnosis of necrotizing fasciitis due to *Pseudomonas aeruginosa* and *Klebsiella pneumonia* infection. Courses of broad-spectrum antibiotics, surgical debridement, and superficial skin grafting were applied with successful effect during ongoing eculizumab therapy. This case highlights the point that it is important to maintain treatment of underlying disorders such as PNH in the presence of life-threatening infections like NF.

## 1. Introduction

Paroxysmal nocturnal hemoglobinuria (PNH) is very rare condition where uncontrolled complement activity leads to systemic complications, principally through intravascular hemolysis and platelet activation. It arises through a somatic mutation of the phosphatidylinositol glycan-A (PIG-A) gene in bone marrow stem cells [1–3], with subsequent disruption to glycosylphosphatidylinositol (GPI) biosynthesis [4] and deficiency of all GPI-anchored cell membrane proteins [5–7]. In particular, deficiency of the complement regulatory proteins, CD55 and CD59, results in increased complement sensitivity of PNH cells, intravascular hemolysis, promotion of inflammatory mediators, and systemic hemoglobin release [8].

Major thrombotic events and a mildly increased susceptibility to infection also feature in PNH. Thrombosis in PNH is also seen during infection and may be partly caused by the increased release of cytokines that promote thromboses. Although infection is not a prominent feature of the disease it does indicate the importance of specific membrane-bound proteins in regulating complement activation and deposition on host cells [9, 10]. Recently, Ge et al. have published the retrospective analysis of 70 patients with PNH. They have documented the prognostic risk factors on survival of patients with PNH as development of thrombotic events, progression to myelodysplastic syndrome or acute myelogenous leukemia (MDS/AML), and recurrent infections [11]. Although there are no surrogate markers supporting that patients with PNH are more prone to infections, one might assume that the infections may be severe and fatal in those patients.

Necrotizing fasciitis (NF) is a group of life-threatening soft tissue infections characterized by widespread necrosis of subcutaneous fascia and tissues, with relative sparing of skin and muscle [12, 13]. The causative bacteria are usually toxin-producing and are extremely virulent [14]. The principle anatomical sites affected are the extremities, trunk, and perineum [15].

We present the case of an elderly patient with aggressive NF while he was followed up for a diagnosis of PNH.

## 2. Case Report

The patient was a 65-year-old male who had not previously experienced any known chronic disease and has been hospitalized in an another medical center on July 2014 for pain, bruises, and wounds on both legs. Skin examination was compatible with petechial rashes, ecchymosis with a maximum width of 20 cm, and necrotic ulcers on both legs (width 4-5 cm; depth 2 cm on the inner face).

Laboratory analysis revealed pancytopenia (hemoglobin 8.4 g/dL; leukocytes 3.0 × 10^9^/L; platelet count 42000/mm^3^) with no other hematological, serologic, coagulation, or biochemical alterations except for lactase dehydrogenase activity of 328 U/L (N: 0–248 U/L). Antinuclear antibody was reported as positive (1/320 granular + 1/160 cytoplasmic). The antinuclear profile was negative. No antiphospholipid antibodies were detected. Since the patient has cytopenias, bone marrow aspiration and trephine biopsies were performed to exclude aplastic anemia and myelodysplastic syndrome and revealed normal findings.

Microthrombi were revealed in both arterial and venous superficial systems in the patient's legs, as evaluated by arterial and venous Doppler ultrasonography, and flow cytometric testing for PNH was conducted. Skin biopsy from border of wounds was planned by surgery department but it was not performed due to potential risk of spreading of infection to healthy tissues. Analysis of CD59 antibodies on antiglycophorin A (CD235a) gated erythrocytes was used to identify erythrocyte PNH cone size. In white blood cells, one GPI-linked protein deficiency in addition to FLAER (FLuorescent AERolysin) was used to demonstrate PNH clones. The deficiency of FLAER/CD24 on CD15+ gated granulocytes and the deficiency of FLAER/CD14 on CD64+ gated monocytes were analysed. Erythrocyte CD59 deficiency, monocyte FLAER/CD14 deficiency, and granulocyte FLAER/CD24 deficiency were shown as 53%, 70%, and 64%, respectively, in flow cytometric PNH analysis.

Intravenous eculizumab therapy was started immediately at a dose of 600 mg weekly for 4 weeks (loading phase), followed by maintenance treatment 1 week later at a dose of 900 mg fortnightly, as indicated at the end of July 2014. Since eculizumab therapy may increase the risk for infections caused by encapsuled bacteria such as* Neisseria meningitidis*, the patient was vaccinated against these pathogens before starting the eculizumab treatment. During follow-up, fulminant tissue destruction was observed, and purulent discharge was observed to drain from the lesions. The lesions progressed to necrotic patches with clear-cut borders and involved subcutaneous tissue and muscles. A diagnosis of NF was made at this point.* Pseudomonas aeruginosa* and* Klebsiella pneumonia* were isolated from wound cultures performed based on lesion samples.

**Figure 1:**
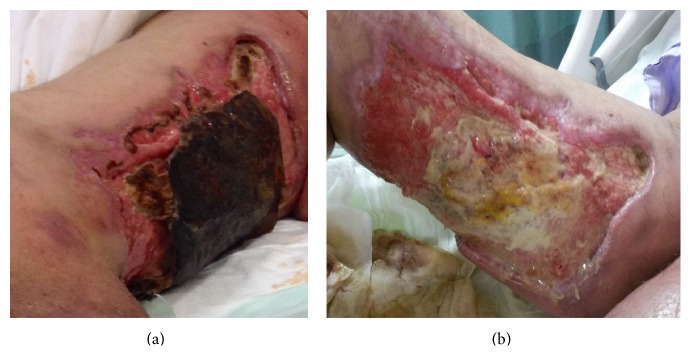
Lesions (a) at first arrival and (b) after debridement.

**Figure 2:**
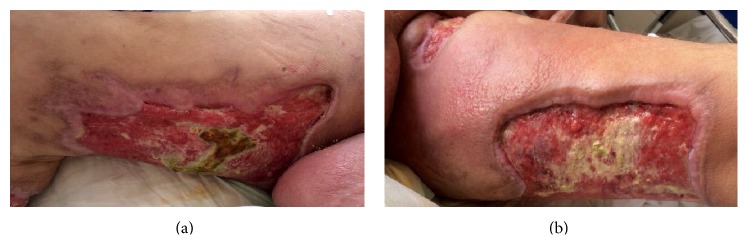
Lesions after infection was controlled with appropriate antibiotherapy and treatment of PNH. (a) Anteromedial part of upper right leg with newly developing scar tissue in the border of wound; (b) interior site of upper left leg showing successful debridement.

**Figure 3:**
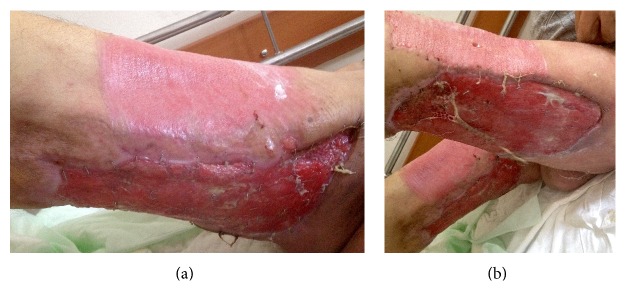
Lesions after skin grafting showing clean grafting site and successful surgery. (a) After skin grafting applied to anteromedial part of right upper leg, showing successful attachment; (b) successful healing of lesion located at posterolateral part of left upper leg.

The patient was referred to our hospital for advanced therapy (Figure 1(a)). Eculizumab was continued at a dose of 600 mg once a week as recommended for the long-term treatment of PNH patients, broad-spectrum antibiotic therapy was started, and general and plastic reconstructive surgery consultations were arranged. Debridement of the raw area over the bilateral lower extremity was conducted (Figure 1(b)). After infection was under control and granulation tissue was seen to form (Figure 2), superficial skin grafting was applied on the bilateral lower extremity. Grafting and antibiotic therapy provided marked recovery in the legs resulting from a multidisciplinary approach involving hematological care, general surgery, and reconstructive surgery (Figure 3).

**Figure 4:**
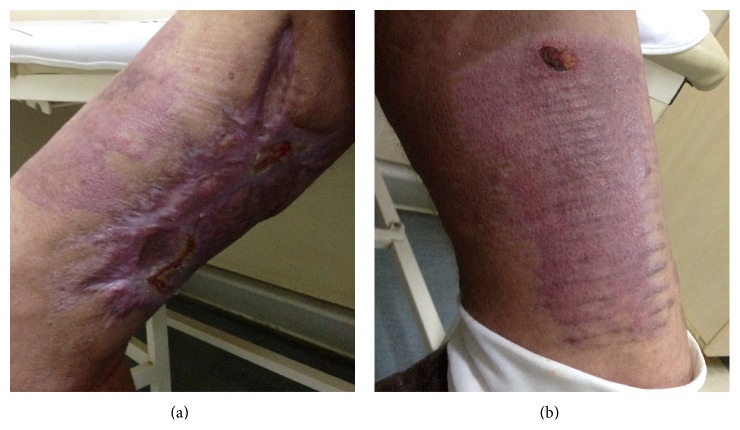
Lesions at time of last control showing almost full healing. Almost totally resolved lesion in (a) right and (b) left upper leg.

The patient was discharged after 50 days of inpatient care. At final follow-up the patient was in remission with good graft attachment and could walk with the help of a walker (Figure 4).

## 3. Discussion

Thrombosis may occur at any site in PNH. For reasons still unknown the most common sites include the intra-abdominal and cerebral veins, making thrombosis a leading cause of morbidity as well as mortality. Thrombosis can also occur at unusual sites including the dermal vessels. Painful discolored skin lesions result when dermal veins are affected, which in rare cases can ulcerate. A separate condition resembling purpura fulminans, which affects larger areas of skin with necrosis, can develop in PNH [16]. In the case reported here we showed that necrotic ulcers were caused by superficial dermal vessel thrombi that progressed to NF.

Previous studies have shown that patients with NF tend to have a number of underlying medical comorbidities or risk factors such as diabetes mellitus, underlying malignancy, smoking, intravenous drug use, renal impairment, and/or obesity [17–19]. Patients with PNH have an increased risk of infections due to complement-associated defects subsequent to CD59 deficiency. The causative agents of NF vary and include two main categories: polymicrobial (type 1) and group A streptococcal (type 2) infections. Our patient's wound culture grew* Pseudomonas aeruginosa* and* Klebsiella pneumonia*.

NF due to* Pseudomonas* infection is a rare but life-threatening disease [20]. It represents a surgical emergency as survival and outcomes depend upon prompt surgical debridement and/or fasciotomy of the infected tissue as well as appropriate antimicrobial and supportive therapy, such as treatment for organ failure [12, 15, 21].

Preventing thrombosis is an important aim in the management of patients with PNH. Eculizumab, a humanized monoclonal antibody, inhibits the terminal complement cascade by binding to human complement protein C5, thereby inhibiting the formation of proinflammatory, prothrombotic C5a and C5b, with subsequent inhibition of membrane attack complex assembly [22, 23]. A series of multinational clinical trials has demonstrated that eculizumab therapy leads to rapid and clinically significant reduction in intravascular hemolysis, is well tolerated, and provides substantial benefits in terms of clinical outcomes [22–28]. Data for patients treated at the National PNH Centre in Leeds, UK, have recently been published that support a continuing dramatic reduction in thrombosis rate, and this is perhaps one of the important factors behind the significantly improved survival for patients treated with this agent [29]. Indeed, the development of any thrombosis in a patient with PNH is now considered one of the primary indicators for the commencement of eculizumab therapy, which should be implemented without delay.

One should not forget that eculizumab therapy may increase the risk for infections caused by encapsuled bacteria such as* Neisseria meningitidis* and the patient must be vaccinated against these pathogens before starting the eculizumab treatment as in our patient. On the other hand, since eculizumab treatment may increase the risk for encapsulated bacteria, increased tendency to infections with* Pseudomonas aeruginosa* and* Klebsiella pneumonia* is not expected in the course of eculizumab therapy. The latter is emphasized by the fact that our patient improved with antibiotic therapy despite continuation of eculizumab.

Since the complement blockade by eculizumab may trigger NF, also in view of the fact that complement may be implicated in the defense against invasive* Streptococcus* [30, 31], there is no direct relationship between NF and PNH in our case, and the important point in this case is to continue the treatment of both conditions concomitantly, since PNH could be the reason underlying the deterioration of the patient's status with NF. Since we started the eculizumab therapy after clinical presentation of NF and the patient improved after starting eculizumab therapy although he had severe wounds, one could argue that eculizumab might have no negative effect on wound healing and treatment of coexisting infections.

Whether a patient has been diagnosed with PNH or not, in the presence of life-threatening infections like NF one should remember that the treatment of underlying disorders is mandatory for saving that patient's life. In particular, PNH should be considered in cases with a slight increase in LDH in the context of mild-to-moderate cytopenia, and tests for differential diagnoses should be performed.
